# Trends and Disparities in Waterpipe Tobacco Smoking Among US Adolescents and Adults: PATH Study 2013-2021

**DOI:** 10.1177/1179173X241275352

**Published:** 2024-08-29

**Authors:** Mohammad Ebrahimi Kalan, Wei Li, Olatokunbo Osibogun, Rime Jebai, Prem Gautam, Olufemi Erinoso, Seyede Yasaman Alemohammad, Sheida Khosravaniardakani, Ghader Dargahi Abbasabad, Raed Behaleh, Kenneth D. Ward, Zoran Bursac, Ziyad Ben Taleb

**Affiliations:** 1 6040 Eastern Virginia Medical School, Norfolk, VA, USA; 2 Department of Psychiatry, 12228 Yale School of Medicine, Yale University, New Haven, CT, USA; 3 Department of Epidemiology, Robert Stempel College of Public Health, 5450 Florida International University, Miami, FL, USA; 4 Department of Health Law, Policy, and Management, 1846 Boston University School of Public Health, Boston, MA, USA; 5 49259 Texas State Board of Pharmacy, Austin, TX, USA; 6 Department of Health Behavior, Policy, and Administration Science, School of Public Health, 6851 University of Nevada, Reno, NV, USA; 7 Department of Social and Behavioral Science, 6889 Virginia Commonwealth University, Richmond, VA, USA; 8 School of Health Sciences, 1651 Baldwin Wallace University, Berea, OH, USA; 9 Health Science Center, 12289 University of New Mexico, Albuquerque, NM, USA; 10 Department of Biostatistics, Robert Stempel College of Public Health, 5450 Florida International University, Miami, FL, USA; 11 Department of Kinesiology, College of Nursing and Health Innovation, 16167 University of Texas at Arlington, Arlington, TX, USA

**Keywords:** waterpipe tobacco smoking, population assessment of tobacco and health, adolescents, adults

## Abstract

**Background:**

Waterpipe tobacco smoking (WTS) is a popular mode of nicotine delivery among young people. We examined the trends and disparities in WTS from 2013 to 2021 among US adolescents and adults.

**Methods:**

Data were from Wave 1 (initially conducted among 32 320 adults and 13 651 adolescents) to Wave 6 (2013-2021) of the Population Assessment of Tobacco and Health Study. We assessed the weighted prevalence of ever and current (past 30-day) WTS for adults and adolescents across waves stratified by demographics.

**Results:**

From 2013-2021 among adolescents, the prevalence of ever and current WTS decreased by 86.5% (7.4% to 1.00%; *p* = 0.0364) and 97.1% (1.65% to 0.05%; *p* = 0.0012), respectively. Despite the decreasing trends among adolescents across all waves, females had a higher prevalence of ever and current WTS compared to males (*p’s* < 0.001 for all trends). Hispanics had the highest prevalence of ever WTS compared to other races/ethnicities (*p’s* < 0.001). Adolescents aged 15-17 had a higher (except Wave 6) prevalence of ever and current WTS than 12-14 years old (*p’s* < 0.001). For adults, the prevalence of ever WTS increased by 27.4% (16.39% to 20.92%; *p *= 0.0006), and current WTS decreased by 45.5% (2.19% to 1.24%; *p* = 0.0012). Young adults aged 18-24 experienced increasing trends in WTS and had the highest prevalence of ever and current WTS compared to other age groups (*p’s* < 0.001) across all waves.

**Conclusions:**

Our study indicates a notable decrease in adolescent WTS prevalence from 2013 to 2021 but an increase of ever WTS among adults. Demographic differences underscore disparities in WTS, calling for tailored interventions.

## Introduction

Globally, waterpipe tobacco smoking (WTS) is a popular mode of nicotine consumption among young people.^1^ In 2023, an estimated 290 000 high school and middle school students in the United States (US) were current (past 30-day) waterpipe tobacco smokers.^2^ Moreover, according to the 2021 National Health Interview Survey’s report, the percentage of current waterpipe users (referred to as regular pipe, waterpipe, or hookah altogether) among adults was 0.9%, with the highest prevalence in the age group 18-44 years old (1.5% for 18-24 years old and 1.6% for 25-44 years old) compared to older adults.^3^ Given the potential health risks associated with WTS and its popularity among young people, it is crucial to examine the trends and disparities in this form of tobacco use to inform targeted prevention and intervention efforts.

Waterpipe tobacco exposes users to the same toxic chemicals found in cigarettes, such as nicotine, carbon monoxide, and polycyclic aromatic hydrocarbons.^1,4-6^ However, due to the longer smoking session duration of WTS (∼1 hour) compared to cigarette smoking, waterpipe smokers can inhale 150-200 times the volume of smoke from a cigarette; thus, they may be exposed to more harmful chemicals than cigarette smokers.^7,8^ Additionally, WTS also causes detrimental health problems such as cancer and heart diseases^5^ and can lead to nicotine dependence faster than cigarette smoking.^9^ Recent research suggests that WTS may serve as a “gateway” to cigarette smoking initiation among young people.^10^ Taken together, the spread of WTS is a critical public health concern and is likely to contribute to increasing tobacco-related morbidity and mortality in the US and beyond.

The popularity of WTS derives from several contributing factors such as the introduction of appealing flavors, the rise of café culture, social media advertising, the perception of reduced harm compared to cigarettes, and, more importantly, the lack of WTS-specific policies and regulations.^10-13^ These influences may vary based on factors like age, sex, and race/ethnicity, leading to differing prevalence rates of WTS. For instance, among US middle school students, Hispanic females showed a higher prevalence of past 30-day WTS compared to other groups,^14^ while among young adults, non-Hispanic Whites exhibited the highest prevalence of current WTS.^15^ Despite the increasing popularity of WTS, particularly among young people,^4,16^ there is a lack of comprehensive national-level data on trends in WTS prevalence across different sociodemographic groups in the US. The broad definition of waterpipe use in the national survey,^3^ which includes regular pipes, distorts the actual prevalence of WTS. The Population Assessment of Tobacco and Health (PATH) Study provides a unique opportunity to examine trends in WTS across multiple waves, using a nationally representative sample and direct measures of WTS.^17^ By assessing WTS trends among adolescents and adults, stratified by age, sex, and race/ethnicity, this study fills a critical gap in our understanding of the evolving WTS landscape in the US. The findings can inform targeted interventions and regulatory efforts to address health disparities and curb the rising popularity of WTS, especially among vulnerable populations. This study specifically assessed the trends in ever and current WTS from 2013 to 2021 among US adolescents and adults by age, sex, and race/ethnicity, using the PATH data.

## Materials and Methods

### Data Source and Study Sample

Data were drawn from the PATH study, an ongoing, nationally representative, longitudinal cohort study of noninstitutionalized civilians aged 12 years and older in the US (see Supplemental Table 1 for study sample details).^17^ The PATH sample includes 45 971 adolescents and adults in Wave 1, with an additional 6065 replenishment sample adults and 3739 replenishment sample adolescents in Wave 4. The PATH study used audio computer-assisted self-interviews in English and Spanish to collect self-report information on tobacco use patterns and associated health behaviors. Adolescents and adults were sampled separately. Children aged 12 to 17 were allowed in the study with parental permission, and assent was also obtained from the adolescents. All emancipated adolescents and adults aged 18 and older provided informed consent. Further details regarding the PATH Study can be found elsewhere.^17^ In the present study, we assessed the weighted prevalence of ever and current WTS across the 6 waves (2013-2021) among 13 651 adolescents (aged 12-17 years) and 32 320 adults (aged ≥18 years). Weighted numbers are presented in Supplemental Tables 1 and 2 The inclusion criteria encompassed respondents who provided information on ever or past 30-day WTS. We included all participants aged 12 years and older as defined by the PATH Study who had complete data on the variables of interest. No specific exclusion criteria were applied beyond the inherent sampling framework of the PATH Study,^17^ as we aimed to maintain the nationally representative nature of the dataset. This current study used publicly available de-identified data and therefore is exempt from Institutional Review Board (IRB) review.

### Study Measures

#### Ever and Current WTS

At each wave, adolescents and adult participants were asked, “Have you ever smoked tobacco in a hookah, even 1 or 2 puffs?” The participants who responded “Yes” were considered ever waterpipe smokers. Participants who were classified as ever waterpipe smokers and had smoked waterpipe in the past 30 days were classified as current waterpipe smokers.^18^

#### Demographic Variables

Demographic variables included age, sex (male, female), and race/ethnicity (Non-Hispanic White, Non-Hispanic Black, Hispanic and Non-Hispanic Others). We stratified age (12-14 years, 15-17 years for adolescents and 18-24 years, 25-44 years, 45-64 years, ≥65 years for adults) to provide a clear picture of WTS based on age groups.^18^

### Data analysis

All analyses were weighted to estimate population distributions and adjust for oversampling and nonresponse.^17,18^ We used the individual replicate cross-sectional weights with 100 balanced replicate weights provided by the PATH study for the 6 waves to account for PATH’s complex survey design with Fay’s adjustment set to 0.3. The replicate weights increase estimate stability with 95% confidence intervals of ever and current WTS to represent the US adolescent and adult populations.^17,18^ We conducted linear regression trend analyses to examine the overall changes in ever and current WTS among adolescents and adults over time.^19^ Furthermore, we assessed the weighted prevalence of ever and current WTS stratified by age, race/ethnicity, and sex. Analyses were conducted using STATA 16 and SAS version 9.4 software, and a *p *<0.05 was considered statistically significant.

This is a cross-sectional comparison, and some participants may have responded in 1 wave but not the other. According to the PATH Public User Guide,^17^ although the PATH Study was not primarily designed for cross-sectional estimation and comparisons, the variance calculations were correctly specified in our study to account for the correlation between the overlapping sets of respondents across waves. Therefore, the estimates from these cross-sectional comparisons can be considered appropriate approximations similar to previous studies using PATH data cross-sectionally over time.^20-26^ As recommended by PATH,^17^ proper variance estimation techniques (eg, resampling methods like balanced repeated replication [BRR]) were employed to account for the complex sample design and correlation between groups across waves, avoiding incorrect inferences from overlapping confidence intervals that neglect the study’s design complexities. This rigorous approach ensures valid cross-wave comparisons and robust findings.^17^

## Results

### Adolescents

**Figure 1.**
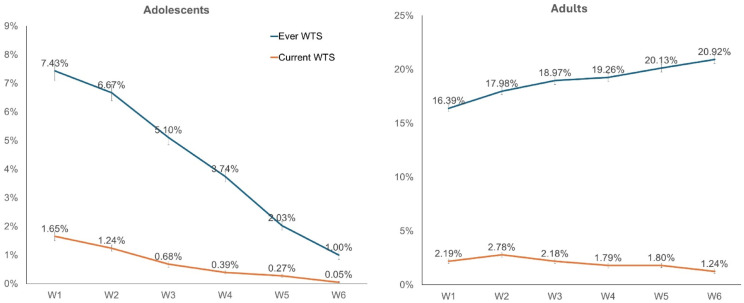
Weighted prevalence of ever and current ^a^WTS among US adolescents (A) and Adults (B). Population Assessment of Tobacco and Health Study, 2013-2021. Abbreviation: ^a^WTS, Waterpipe tobacco smoking; W, wave. Note: Error bars indicate 95% confidence intervals for corresponding percentages.

From 2013 to 2021, the prevalence of ever WTS among US adolescents gradually decreased from 7.43% to 1.00%, with a similar decline in current use from 1.65% to 0.05% (*p’s* < 0.05 for all trends; Figure 1(A)). Within age groups, older adolescents (15-17 years; except for wave 6 in current WTS) consistently exhibited higher rates of WTS (both ever and current) compared to younger ones (12-14 years), though both groups generally experienced declines over time. Females consistently demonstrated slightly higher rates of WTS compared to males across waves, with fluctuations in prevalence observed during waves 4 and 5 in current WTS and wave 6 in ever WTS. Hispanic adolescents had the highest rates of ever WTS compared to Non-Hispanic White adolescents over time. Non-Hispanic Black adolescents had lower rates of ever WTS in Waves 1-3 and 5 compared to other race groups.

### Adults

From 2013-2021, the overall prevalence of ever WTS (16.39% to 20.92%) significantly increased (*p’s* < 0.001 for all trends). There was no significant difference during the same period for the overall prevalence of current WTS (from 2.19% to 1.24%; Figure 1(B)). During the same period, young adults (aged 18-24) had the highest prevalence of current WTS, compared to the older age groups (*p*-values <0.001) (Table 1). Additionally, we found a high proportion of ever and current WTS among males compared to females (*p*’s < 0.001). Racial patterns showed that non-Hispanic Whites had the lowest prevalence of current WTS compared to Hispanics, non-Hispanic Blacks, and other race groups over time (*p*’s < 0.001).

**Table 1.** Weighted Prevalence of ^a^WTS Stratified by Age, Gender, and Race Among US Adolescents and Adults: Population Assessment of Tobacco and Health Study, 2013-2021.

Characteristic	Wave1 (2013-2014)		Wave2 (2014-2015)		Wave3 (2015-2016)	
	Ever WTS	Current WTS	Ever WTS	Current WTS	Ever WTS	Current WTS
	Weighted % (^a^95% CI)	Weighted % (95% CI)	Weighted % (95% CI)	Weighted % (95% CI)	Weighted % (95% CI)	Weighted % (95% CI)
**Adolescents overall**	7.43 (6.78-8.13)	1.65 (1.37-1.99)	6.66 (6.12-7.25)	1.24 (1.05-1.47)	5.10 (4.63-5.63)	0.68 (0.51-0.90)
**Age** (**years)**						
12-14	2.01 (1.68-2.40)	0.46 (0.32-0.66)	1.98 (1.60-2.44)	0.40 (0.26-0.60)	1.16 (0.89-1.51)	0.19 (0.10-0.35)
15-17	12.95 (11.81-14.18)	2.86 (2.36-3.46)	11.48 (10.56-12.47)	2.11 (1.77-2.51)	9.11 (8.25-10.05)	1.18 (0.88-1.59)
**Sex**						
Male	7.10 (6.45-7.82)	1.39 (1.10-1.75)	6.16 (5.53-6.84)	1.06 (0.82-1.38)	4.57 (4.04-5.17)	0.67 (0.46-0.97)
Female	7.77 (6.83-8.83)	1.92 (1.53-2.41)	7.18 (6.40-8.04)	1.44 (1.16-1.77)	5.64 (4.90-6.49)	0.70 (0.48-1.00)
**Race**						
Non-hispanic White	7.40 (6.64-8.24)	1.78 (1.46-2.17)	6.49 (5.83-7.22)	1.20 (0.92-1.58)	4.82 (4.19-5.54)	0.71 (0.49-1.01)
Non-hispanic Black	4.82 (3.81-6.09)	1.31 (0.86-2.00)	5.07 (4.01-6.39)	0.95 (0.55-1.65)	4.18 (3.20-5.44)	0.84 (0.43-1.64)
Hispanic	9.37 (8.18-10.71)	1.69 (1.20-2.38)	8.64 (7.35-10.14)	1.78 (1.25-2.53)	6.88 (5.70-8.28)	0.70 (0.43-1.13)
Non-hispanic other	6.86 (5.38-8.72)	1.27 (0.67-2.40)	6.07 (4.62-7.94)	0.94 (0.47-1.84)	5.00 (3.86-6.45)	0.48 (0.20-1.13)
**Adults overall**	16.39 (15.76-17.03)	2.19 (2.03-2.37)	17.98 (17.32-18.66)	2.78 (2.60-2.98)	18.97 (18.25-19.71)	2.18 (2.02-2.36)
**Age** (**years)**						
18-24	44.36 (42.26-46.48)	10.73 (9.78-11.76)	46.98 (44.99-48.98)	13.00 (12.11-13.95)	46.41 (44.22-48.60)	9.19 (8.31-10.15)
25-44	22.90 (21.61-24.24)	2.03 (1.77-2.34)	26.30 (24.90-27.75)	2.91 (2.59-3.27)	29.29 (27.75-30.87)	2.42 (2.10-2.78)
45-64	6.77 (6.27-7.32)	0.28 (0.19-0.42)	7.46 (6.89-8.08)	0.37 (0.26-0.54)	8.06 (7.45-8.71)	0.56 (0.42-0.77)
≥65	2.48 (1.99-3.09)	0.03 (0.01-0.18)	2.87 (2.31-3.56)	0.09 (0.03-0.29)	3.12 (2.53-3.85)	0.19 (0.07-0.47)
**Sex**						
Male	19.54 (18.72-20.38)	2.69 (2.44-2.94)	21.43 (20.55-22.33)	3.31 (3.03-3.62)	22.44 (21.48-23.42)	2.58 (2.33-2.86)
Female	13.47 (12.86-14.11)	1.74 (1.55-1.95)	14.83 (14.17-15.52)	2.29 (2.06-2.55)	15.81 (15.13-16.51)	1.82 (1.61-2.05)
**Race**						
Non-hispanic White	15.42 (14.60-16.27)	1.60 (1.43-1.78)	16.69 (15.81-17.61)	1.88 (1.68-2.10)	17.36 (16.43-18.33)	1.37 (1.20-1.56)
Non-hispanic Black	15.00 (13.72-16.38)	2.79 (2.27-3.43)	18.58 (17.05-20.22)	4.97 (4.26-5.80)	20.34 (18.75-22.03)	3.94 (3.30-4.70)
Hispanic	19.47 (18.23-20.77)	3.88 (3.41-4.42)	21.31 (19.73-22.97)	4.43 (3.79-5.17)	22.90 (21.23-24.67)	3.52 (3.02-4.10)
Non-hispanic other	20.79 (18.72-23.02)	3.07 (2.42-3.90)	23.04 (20.86-25.37)	4.08 (3.31-5.03)	23.79 (21.36-26.40)	3.62 (2.70-4.84)

Characteristic	Wave4 (2016-2018)		Wave5 (2018-2019)		Wave6 (2021)	
	Ever WTS	Current WTS	Ever WTS	Current WTS	Ever WTS	Current WTS
	Weighted % (95% CI)	Weighted % (95% CI)	Weighted % (95% CI)	Weighted % (95% CI)	Weighted % (95% CI)	Weighted % (95% CI)
**Adolescents overall**	3.74 (3.37-4.14)	0.39 (0.30-0.51)	2.03 (1.75-2.36)	0.27 (0.18-0.40)	1.00 (0.74-1.35)	0.05 (0.02-0.16)
**Age (years)**						
12-14	0.80 (0.62-1.03)	0.08 (0.03-0.18)	0.44 (0.29-0.68)	0.13 (0.05-0.32)	0.52 (0.21-1.31)	0.06 (0.01-0.42)
15-17	6.75 (6.07-7.49)	0.70 (0.54-0.92)	3.66 (3.14-4.26)	0.41 (0.27-0.64)	1.18 (0.87-1.60)	0.05 (0.02-0.18)
**Sex**						
Male	3.73 (3.29-4.24)	0.39 (0.27-0.56)	1.97 (1.63-2.38)	0.27 (0.15-0.48)	1.00 (0.70-1.44)	0.03 (0.004-0.18)
Female	3.74 (3.22-4.34)	0.38 (0.25-0.59)	2.11 (1.74-2.55)	0.26 (0.16-0.44)	0.98 (0.64-1.50)	0.08 (0.02-0.29)
**Race**						
Non-hispanic Bhite	3.26 (2.80-3.79)	0.32 (0.21-0.49)	1.72 (1.36-2.17)	0.17 (0.08-0.39)	0.47 (0.25-0.86)	0.03 (0.004-0.21)
Non-hispanic Black	3.59 (2.74-4.68)	0.72 (0.43-1.21)	1.39 (0.85-2.27)	0.22 (0.06-0.81)	1.70 (0.93-3.09)	0.16 (0.03-0.94)
Hispanic	5.65 (4.77-6.69)	0.44 (0.24-0.79)	3.33 (2.60-4.24)	0.59 (0.32-1.08)	1.62 (1.06-2.47)	0.08 (0.01-0.44)
Non-hispanic other	2.77 (1.95-3.93)	0.31 (0.12-0.76)	1.64 (0.99-2.71)	0.04 (0.004-0.41)	1.51 (0.77-2.93)	NA
**Adults overall**	19.26 (18.54-20.00)	1.79 (1.65-1.93)	20.13 (19.41-20.87)	1.80 (1.62-2.00)	20.92 (20.15-21.72)	1.24 (1.11-1.38)
**Age (years)**						
18-24	40.65 (38.61-42.73)	6.54 (5.94-7.19)	33.73 (31.98-35.52)	5.11 (4.53-5.75)	23.43 (22.20-24.71)	2.75 (2.34-3.22)
25-44	31.48 (29.98-33.01)	2.32 (2.06-2.61)	36.22 (34.73-37.73)	2.96 (2.55-3.42)	40.95 (39.21-42.71)	2.35 (2.00-2.76)
45-64	8.48 (7.89-9.12)	0.53 (0.39-0.72)	9.59 (8.92-10.31)	0.58 (0.41-0.81)	10.77 (9.92-11.68)	0.37 (0.24-0.56)
≥65	3.27 (2.70-3.95)	0.05 (0.01-0.20)	3.75 (3.14-4.47)	0.04 (0.01-0.20)	4.81 (4.09-5.65)	0.03 (0.02-0.25)
**Sex**						
Male	22.56 (21.64-23.50)	1.95 (1.75-2.17)	23.22 (22.30-24.17)	1.97 (1.73-2.25)	23.88 (22.83-24.97)	1.23 (1.05-1.44)
Female	16.24 (15.50-17.01)	1.64 (1.45-1.85)	17.28 (16.52-15.07)	1.65 (1.45-1.87)	18.21 (17.42-19.02)	1.24 (1.06-1.46)
**Race**						
Non-hispanic White	17.56 (16.60-18.57)	1.09 (0.95-1.26)	18.09 (17.12-19.10)	0.78 (0.65-0.95)	18.61 (17.55-19.72)	0.46 (0.36-0.58)
Non-hispanic Black	22.04 (20.69-23.45)	3.91 (3.40-4.49)	24.16 (22.84-25.54)	4.89 (4.16-5.72)	26.27 (24.72-27.89)	4.33 (3.69-5.07)
Hispanic	23.52 (21.86-25.26)	2.77 (2.35-3.26)	24.37 (22.52-26.32)	3.02 (2.47-3.69)	25.56 (23.61-27.62)	1.93 (1.48-2.50)
Non-hispanic other	22.44 (20.39-24.63)	2.54 (1.86-3.47)	23.75 (21.69-25.94)	3.13 (2.33-4.20)	23.93 (21.23-26.85)	1.43 (0.97-2.11)

Abbreviation: WTS, Waterpipe tobacco smoking.

^a^95%CI, 95% confidence interval, Note: Percentages (%) and 95% CIs are weighted to represent the US population. NA = no data was available for this cell.

## Discussion

This study is among the first to report the prevalence of ever and current WTS using trend analysis from a representative sample of the US population across age, sex, and race/ethnicity. Among US adolescents, the estimated prevalence of ever and current WTS significantly decreased by about 87% and 97% from 2013 to 2021, respectively. During the same period, ever and current WTS were more common among older adolescents (15-17 years). Furthermore, ever WTS was more common among females than males and current WTS was more common among Hispanics than other races. The rate of current WTS among adults decreased by 46%, suggesting a decline in regular use during the study period. Despite this decrease in ongoing usage, there was a 22% increase in the prevalence of ever WTS, indicating heightened curiosity and experimentation with WTS among adults. Furthermore, patterns of both ever and current WTS were more prevalent among males and younger adult age group (18-24 years), and notably less common among non-Hispanic Whites. This demographic variability underscores the need for targeted public health strategies that consider gender, age, and race/ethnicity differences in WTS behavior.

We observed notable differences in the trends of ever and current WTS between adolescents and adults. Adolescents typically lack access to waterpipe lounges and may find the handling and operation of waterpipe devices challenging, as these activities require a certain level of experience and autonomy that adolescents generally do not possess. Additionally, the legal age restrictions and less independence at this developmental stage further restrict their ability to engage in WTS regularly. Conversely, young adults, particularly college students, often find themselves in environments conducive to experimenting with WTS. The autonomy associated with living in dormitories, coupled with the social dynamics of college life and party culture, provides ample opportunity for WTS.^13^ Additionally, this age group’s increased independence from parental supervision and greater integration into social networks that may encourage WTS contribute to the higher prevalence observed in this demographic.

Moreover, the recent global health crisis, such as the COVID-19 pandemic, likely influenced patterns of tobacco use. The restrictions on social gatherings and temporary closures of lounges and social venues may have temporarily decreased the prevalence of WTS among both adolescents and adults, reflecting broader changes in social behavior during the pandemic. Future research should consider these age-specific factors and social environments when investigating the drivers of WTS, focusing on how age-related differences in autonomy and social exposure influence tobacco use behaviors. This approach will provide valuable insights into effective prevention and intervention strategies tailored to distinct age groups.

Among adults, we only found a significant increase in ever WTS, which might be explained by the appealing flavors, misperception of health effects of WTS compared to other tobacco products, and the social nature of this smoking behavior, where smokers like to share the waterpipe with multiple peers.^13^ This increase in ever WTS among adults underscores the need for further research into the factors driving WTS initiation and usage patterns in this demographic, particularly in light of potential bans on menthol cigarettes and flavored cigars. Such bans may prompt transitions among adults towards menthol-flavored WTS, enjoying the minimal regulations of flavor availability.^27,28^ This situation underscores the need to understand the factors influencing tobacco use behaviors in this context, as excluding waterpipe from flavored tobacco sales restrictions could exacerbate health inequities in those groups most prone to nicotine addiction.^27^

The higher prevalence of both ever and current WTS among non-White racial/ethnic groups, particularly among males and younger adults aged 18-24 years, is consistent with previous findings in the literature.^29-31^ As alluded to above, several factors may contribute to this pattern. Firstly, WTS has deep cultural roots in many Middle Eastern and Southeast Asian communities, where it is often viewed as a traditional and socially acceptable practice.^11,31,32^ Additionally, the introduction of flavored tobacco products and the growth of hookah lounges and cafés have made WTS more appealing and accessible to younger demographics, particularly in urban areas with higher representation of racial/ethnic minority groups.^33^ Furthermore, targeted marketing strategies by the tobacco industry, including the portrayal of WTS as a safer and more socially acceptable alternative to cigarettes, may have resonated more strongly with certain racial/ethnic communities.^34^ To address the higher prevalence of WTS among non-White groups, especially males and younger adults, targeted interventions are needed. These interventions should include stricter regulations on flavored tobacco, marketing restrictions, and educational campaigns about health risks in culturally affected communities.^5,11,12,30,32,35^

Our study is not without limitations. First, the assessment of WTS was based on self-reported data. This may under-report WTS due to social desirability bias. However, earlier research^36^ has suggested a correlation between self-reported tobacco use among youth and young adults and biomarkers of tobacco consumption. Second, we only assessed the general demographic variables (age, sex, and race/ethnicity) of WTS from the PATH study. For example, we used public data on PATH for both youth and adults and since the youth sample did not have sexual orientation questions publicly available, future studies may include this important demographic along with other environmental factors such as education and poverty level. Another study limitation was the absence of a priori power analysis. We utilized all available data from the PATH Study on WPT which likely provided sufficient statistical power for our analyses. Despite these limitations, our study provides prevalence estimates for ever and current WTS from an overall representative US population over 9 years. Our study provides insights into the trends and patterns of WTS in the US. While our findings don’t directly address intervention strategies, they contribute to the growing body of knowledge on WTS. Previous research has highlighted the importance of gender-specific education programs, age-appropriate social media campaigns, and culturally sensitive community outreach as potential strategies for addressing WTS.^11,30,31,34,37-41^ For instance, theory-based health education programs have shown promise in WTS cessation for women in the Middle East,^42^ while community-based approaches involving religious leaders have effectively reached ethnic minorities in different jurisdictions.^43-45^ Future research could explore how our findings on WTS trends might inform these approaches, particularly in developing mobile health (mHealth) interventions and peer-led education programs that leverage widespread mobile technology use and peer influence. Additionally, further studies could investigate how the patterns we’ve observed might contribute to the development of comprehensive, culturally tailored policies and programs aimed at reducing WTS prevalence and its associated health risks globally, with a particular focus on young people.

## Conclusions

From 2013 to 2021, adolescent WTS declined, with older adolescents and Hispanic adolescents showing higher rates. Conversely, adult ever WTS prevalence increased, particularly among young adults, with males and Hispanic adults exhibiting higher rates. These findings may inform subsequent public health surveillance as the tobacco landscape continues to evolve in the US. Furthermore, the findings may serve as a foundation for future research to identify reasons for the observed demographic variations and to develop interventions to reduce WTS among targeted populations in the US. Identifying these reasons will be essential for developing effective interventions, particularly for groups disproportionately affected by WTS. Through such targeted research and intervention efforts, public health initiatives can be better tailored to address the specific needs and behaviors of different demographic groups, ultimately reducing the prevalence of WTS and improving overall community health.

## Supplemental Material

Supplemental Material - Trends and Disparities in Waterpipe Tobacco Smoking Among US Adolescents and Adults: PATH Study 2013-2021 Supplemental Material for Trends and Disparities in Waterpipe Tobacco Smoking Among US Adolescents and Adults: PATH Study 2013-2021 by Mohammad Ebrahimi Kalan, Wei Li, Olatokunbo Osibogun, Rime Jebai, Prem Gautam, Olufemi Erinoso, Seyede Yasaman Alemohammad, Sheida Khosravaniardakani, Ghader Dargahi Abbasabad, Raed Behaleh, Kenneth D. Ward, Zoran Bursac, and Ziyad Ben Taleb in Tobacco Use Insights
