# Supplementary material for: Trends and Disparities in Waterpipe Tobacco Smoking Among US Adolescents and Adults: PATH Study 2013-2021
Source: Tob Use Insights. 2024 Aug 29;17:1179173X241275352. doi: 10.1177/1179173X241275352 (PMC11363053; doi:10.1177/1179173X241275352)
Supplement: Supplemental Material - Trends and Disparities in Waterpipe Tobacco Smoking Among US Adolescents and Adults: PATH Study 2013-2021 [file sj-pdf-1-tui-10.1177_1179173X241275352.pdf]

Supplementary Table 1. PATH Study data collection years and numbers (crude and weighted)

Wave (years)	Unweighted Adolescents	Unweighted Adults	Weighted Youth	Weighted Adults
Wave 1 (2013-2014)	13,651	32,320	24,852,363	236,691,585
Wave 2 (2014-2015)	12,172	28,362	24,670,517	238,690,238
Wave 3 (2015-2016)	11,814	28,148	24,784,223	239,802,069
Wave 4 (2016-2018)	14,798*	33,822*	24,804,094	243,641,156
Wave 5 (2018-2019)	12,098	34,309	24,737,765	246,520,211
Wave 6 (2021)	5,652	30,516	15,856,412	248,528,820

of interviews among adolescents and adults by wave (Waves 1-6).

Note: * Wave 4 included 3,739 “replenishment sample adolescents” and 6,065 “replenishment sample adults”.

Supplementary Table 2. Unweighted and weighted prevalence of WTS Stratified by age, gender, and race among US Adolescents and Adults: Population Assessment of Tobacco and Health Study, 2013-2021 (Wave 1&6 only).

Characteristic	Wave1 (2013-2014)		Wave6 (2021)	
	Ever WTS unweighted # (weighted #)	Current WTS unweighted # (weighted #)	Ever WTS unweighted # (weighted #)	Current WTS unweighted # (weighted #)
**Adolescents overall**	1,005 (1,846,340)	226 (409,853)	64 (157,627)	4 (8,524)
**Age (years)**				
12-14	146 (251,884)	37 (57,341)	5 (22,766)	1 (2,473)
15-17	859 (1,594,455)	189 (352,513)	59 (134,861)	3 (6,051)
**Sex**				
Male	497 (905,987)	96 (177,282)	34 (155,724)	1 (2,087)
Female	508 (940,352)	130 (232,571)	29 (75,093)	3 (6,437)
**Race**				
Non-Hispanic White	485 (1,002,925)	124 (241,535)	12 (35,062)	1 (2,087)
Non-Hispanic Black	80 (166,507)	22 (45,310)	11 (32,079)	1 (2,977)
Hispanic	340 (518,419)	62 (93,710)	25 (52,005)	1 (2,473)
Non-Hispanic Other	100 (158,489)	18 (29,299)	10 (24,788)	0 (-)*
**Adults overall**	10,623 (38,785,014)	1,720 (5,188,011)	8,982 (51,870,239)	621 (3,071,931)
**Age (years)**				
18-24	5,061 (13,621,120)	1,261 (3,295,066)	2,117 (6,795,509)	270 (798,837)
25-44	4,183 (18,556,757)	400 (1,648,844)	5,363 (33,757,009)	311 (1,943,913)
45-64	1,160 (5,530,702)	55 (231,258)	1,139 (8,591,081)	35 (291,968)
≥65	218 (1,069,490)	4 (12,843)	363 (2,726,640)	5 (37,213)
**Sex**				
Male	5,826 (22,227,948)	978 (3,051,470)	4,427 (28,435,241)	242 (1,467,277)
Female	4,797 (16,557,066)	742 (2,136,541)	4,549 (23,410,958)	379 (1,604,654)
**Race**				
Non-Hispanic White	6,197 (24,002,142)	797 (2,489,789)	4,596 (28,967,259)	128 (713,999)
Non-Hispanic Black	1,229 (4,098,400)	271 (761,840)	1,378 (7,203,868)	257 (1,190,992)
Hispanic	2,185 (6,900,627)	478 (1,376,774)	1,787 (8,824,140)	139 (667,940)
Non-Hispanic Other	1,012 (3,783,846)	174 (559,608)	814 (4,844,779)	58 (290,673)

***Zero participants were in this cell. Abbreviation: WTS, Waterpipe tobacco smoking;
